# Hirschsprung disease with Edward syndrome: A rare association: A case report

**DOI:** 10.1016/j.ijscr.2021.106084

**Published:** 2021-06-09

**Authors:** Bibek Man Shrestha, Diwan Shrestha, Suraj Shrestha, Anil Bist, Sanjeev Kharel, Dinesh Prasad Koirala

**Affiliations:** a Maharajgunj Medical Campus, Institute of Medicine, Kathmandu, Nepal; b Department of GI and General Surgery, Tribhuvan University Teaching Hospital, Institute of Medicine, Kathmandu, Nepal

**Keywords:** Chromosomal abnormalities, Ethics, Edward syndrome, Trisomy

## Abstract

**Introduction and importance:**

Edward's syndrome (ES) occurs as a result of trisomy of chromosome 18 and is associated with multisystem congenital anomalies. The association of ES with various gastrointestinal malformations but Hirschsprung disease (HD) is well documented.

**Case presentation:**

A female infant on her 5th day of life presented with episodes of bilious vomiting along with abdominal distension and no passage of stool. The child had a small head and prominent occiput, low set abnormal ears, small jaw, upturned nose, widely spaced eyes, small neck with widely spaced nipples, clenched hands with overlapping fingers, flexed big toe, and prominent heels.

**Clinical discussion:**

Edward syndrome is associated with multisystem congenital abnormalities of which gastrointestinal abnormalities make up the most part. The condition can be identified by fetal ultrasound screening. Surgical correction of associated congenital anomalies at different times along with lifelong supportive management is important.

**Conclusions:**

Edward syndrome can present as Hirschsprung disease as a part of associated gastrointestinal Malformation. Often, early identification and termination of the pregnancy in antenatal life can reduce the suffering. Surgical correction of associated anomalies along with supportive care forms the cornerstone of management. However, the prognosis remains poor.

## Highlights

• Association of Edward syndrome (Trisomy 18) with Hirschsprung disease is still not clear.
• Although prognosis is poor, surgical correction of associated anomalies along with supportive care is the crucial in management.

## 1 Introduction

Hirschsprung's disease (HD) is a congenital malformation in which parasympathetic intrinsic ganglion cells in the submucosal and myenteric plexuses of the hindgut are missing [1]. HD has been identified in a large number of chromosomal abnormalities. Down's syndrome/Trisomy 21 is by far the most common chromosomal abnormality associated with HD [2]. Trisomy of chromosome 18, otherwise known as Edward syndrome (ES) results in multisystem malformative condition including major gastrointestinal malformations including esophageal atresia, tracheoesophageal fistula, diaphragmatic hernia, and omphalocele [3]. HD in ES has not been yet reported in the literature. Herein, we report a case of HD with Edward syndrome in an infant. This report has been written in line with SCARE guidelines [4].

## 2 Case report

A Five-day-old female infant, born to a 23-year G_2_P_1_L_1_ mother by spontaneous vaginal delivery at 39^+2^ period of gestation was brought to the emergency department with a complaint of multiple episodes of bilious vomiting along with abdominal distension and unable to pass stool since birth. There was no history of fever, lethargy, inconsolable cry, and irritability. The female had no history of spontaneous abortion and her first child is healthy and normal.

**Fig. 1:**
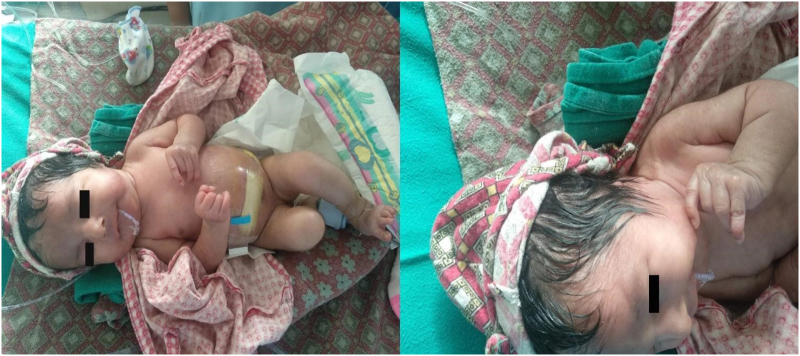
Showing small head, upturned nose, widely spaced eyes, small neck with widely spaced nipples, clenched hands, flexed big toe (left) along with low set ear (right).

On examination, the child was conscious and ill-looking with a small head but prominent occiput, low set abnormal ears, small jaw, upturned nose, widely spaced eyes, small neck with widely spaced nipples, clenched hands with overlapping fingers, flexed big toe, and prominent heels; features suggestive of Edward's syndrome (Fig. 1). She was afebrile but tachycardic (heart rate: 160/min) and tachypneic (respiratory rate-70/min) and other vitals were stable. The abdomen was distended, tense with dilated veins, everted umbilicus, and absent bowel sound. The orogastric tube was thus placed for decompression.

**Fig. 2:**
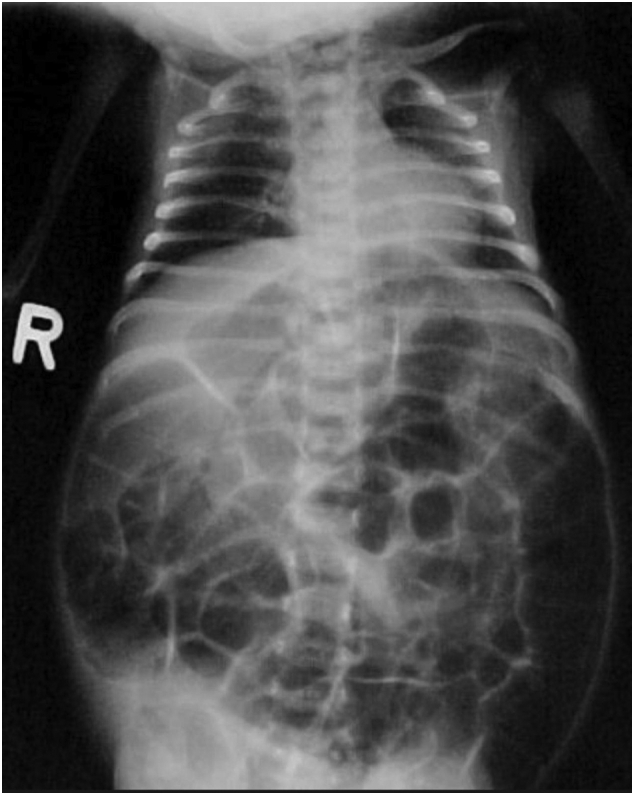
Plain abdominal X-ray supine view showing multiple loops of dilated large bowel suggesting large bowel obstruction.

**Fig. 3:**
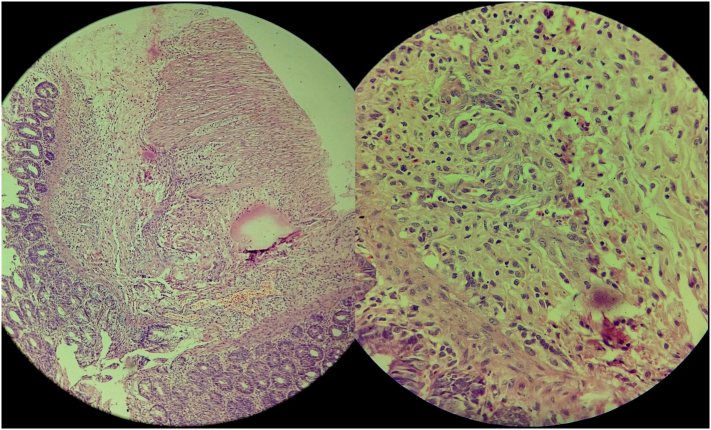
Section (left) showing mucosa, submucosa and muscularis propria of descending colon (H&E, magnification 100×) and section (right) showing mucosa, submucosa of descending colon with absent ganglion cells (H&E, magnification 400×).

All the hematological parameters were within normal limits. X-ray abdomen supine view revealed marked dilatation of large bowel with no gas in the rectum (Fig. 2). USG abdomen and pelvis revealed a gaseous abdomen with minimal interloop ascites. There was no passage of stool even after the rectal wash. Considering the possibility of intestinal obstruction, an exploratory laparotomy was done which revealed multiple bands of approximately 3 cm and 10 cm distal to an ileocolic junction along with malrotation of the gut. Bands were released and the malrotation was corrected. Furthermore, Biopsies were taken from splenic flexure and descending colon which showed the absence of ganglion cells suggestive of HD (Fig. 3). With typical clinical features of ES and the absence of ganglionic cells, a diagnosis of Hirschsprung disease with ES was made. The baby underwent colostomy and her first stage of the Soave procedure for the management of HS is planned at 6 months of age. All the procedures were performed by the experienced team of gastrointestinal surgeons of Tribhuvan University Teaching Hospital and were well tolerated by the patient. Parents were counseled regarding the disease diagnosis and expected long-term outcomes, quality of life, and the prospects of poor prognosis even with the multiple surgical interventions. The patient is under regular follow-up.

## 3 Discussion

Edward syndrome is a rare chromosomal disorder that occurs due to non-disjunction or translocation of chromosome 18 with a live prevalence of 1/3600 to 1/10,000 [5]. Multiorgan involvement includes congenital heart disease in about 90% of the patients, the musculoskeletal system of upper and lower extremities, head and neck, genitourinary system as well as gastrointestinal system [3].

### 3.1 Clinical manifestations

Major clinical manifestations of the syndrome are represented by prenatal growth deficiency, specific craniofacial features in the postnatal period collectively associated with other major and, minor anomalies along with noticeable psychomotor and cognitive developmental delay in later days of life [6]. Among many of the musculoskeletal manifestations associated with Edward syndrome, the child presented with the low sternum, widely spaced breasts, narrow pelvis, dorsiflexion of big toes, fixed flexion deformity of fingers [7]. Our patient presented with minor abnormalities which include widely spaced eyes, a small neck with widely spaced nipples, and clenched hands with overlapping fingers, flexed big toe, and prominent heels.

Of various gastrointestinal abnormalities that can occur with ES as previously mentioned, however, HS is not one of them. Although chromosomal abnormalities account for 12% of the syndromic association of HD, Down's syndrome is the commonest. Other syndromes like multiple congenital anomalies-Mental retardation syndrome, Mowat Wilson Syndrome, DiGeorge Syndrome, Mosaic trisomy 8, XXY chromosomal constitution, partial duplication of chromosome 2q, tetrasomy 9p should be considered in the vicinity [2]. There have been no reports documenting HS association with ES to date.

Presentation of HD in neonates is early with symptoms suggesting intestinal obstruction, marked by distention of the abdomen, the passage of meconium after 48 h, feeding intolerance, and bilious vomiting [8].

### 3.2 Prenatal screening

Anatomical malformations associated with trisomy 18 can be detected by prenatal ultrasound and amniocentesis or cordocentesis tests can be carried out for karyotyping or fluorescent in situ hybridization [9]. Early diagnosis of this syndrome helps the parents to choose between the continuation or termination of the pregnancy considering the probable anomalies that can occur [10]. However, the mother did not have her antenatal scans done and thus the prenatal diagnosis was missed. Though G-band karyotyping of peripheral blood after birth can help to make the diagnosis of ES [11]. In our case, the clinical features in our case were obvious to support the diagnosis, and thus no karyotyping was performed.

### 3.3 Management of HD and Edward syndrome

Surgical intervention with resection of aganglionic bowel is the principal treatment for Hirschsprung disease. Colostomies were performed readily to prevent the development of enterocolitis [12]. Depending on their health status, and severity of colon distension, Hirschsprung disease diagnosed in infants need corrective surgery immediately within the first few weeks or months of life [8]. The same was done in our case with the child planned for corrective surgery after a few months of life. Corrective surgical techniques used include Swenson, Duhamel, and Soave techniques which can be carried out either open or laparoscopically. Despite undergoing corrective surgery, the degree of severity of the associated anomalies determines the long-term prognosis of HD [2]. Such procedure in the neonatal period should be viewed as an acceptable procedure in the management of the infant with Hirschsprung disease and is restricted to infants who are stable and without signs of enterocolitis at the time of the pull-through procedure [13]. ES was previously thought to be lethal and incompatible with life, and medical therapies to prolong life were barely provided. However, many children with this condition are surviving much longer, with reports of individuals living for more than a decade, provided proper medical and surgical interventions, albeit with a wide range of disabilities [14]. This bleak evidence has provided the parents with a hope to provide all the possible treatment to their offspring.

### 3.4 Ethical considerations and counseling

From an ethical point of view, it is necessary for a physician to identify those children who would actually benefit from an interventionist approach, consider factors like overall survival, long term quality of life, and not simply deny an interventionist approach based solely on a diagnosis of trisomy 18. Simultaneously, it is of paramount importance to respect parental values, consider parental decisions, and also the right to parental authority [15]. After making a successful diagnosis of ES prenatally, proper counseling is needed to help the parents make decisions regarding the possible future outcomes of the child [6]. Of all the infants diagnosed with Edward syndrome, only a 5 to 10% survival rate is reported beyond the first year of life, the major cause of death being cardiorespiratory failure which makes the role of proper counseling more crucial [16]. Depending upon the choice of parents, intensive management of Edward syndrome includes supportive treatment of cardiovascular, respiratory systems as well as nutritional support along with the prevention of infection for a better life [16,17]. Our patient was managed for HD and proper counseling was done to the parents about the course of the ES.

## 4 Conclusion

HD can occur with Edward syndrome. More understanding of the association of HD with syndromic diseases including Edward's can provide insights into the condition. Additionally, termination of pregnancy once diagnosed with trisomy 18 as previously thought no longer holds true and all the necessary interventions have to be provided with a consensual decision of treating physician and the parents. Proper counseling at different points of time along with intensive supportive treatment is necessary for optimal survival of the child.

## Consent to participate

Written informed consent was obtained from the patient's parent for publication of this case report and accompanying images. A copy of the written consent is available for review by the Editor-in-Chief of this journal on request.

## Ethical approval

Not required.

## Funding

None.

## Guarantor

Bibek Man Shrestha.

## Research registration number

None.

## CRediT authorship contribution statement

Dinesh Prasad Koirala (DPK), Diwan Shrestha (DS), and Bibek Man Shrestha (BMS) = Study concept, Data collection, and surgical therapy for the patient
BMS and Suraj Shrestha (SS) = Writing - original draft preparation
Anil Bist (AB) and Sanjeev Kharel (SK) = Editing and writing
DPK and DS = senior author and manuscript reviewer.

All the authors read and approved the final manuscript.

## Declaration of competing interest

None to declare.
